# Editorial: Machine learning-assisted diagnosis and treatment of endocrine-related diseases

**DOI:** 10.3389/fendo.2023.1305897

**Published:** 2023-12-18

**Authors:** Heng Zhang, Ulf D. Kahlert, Wenjie Shi

**Affiliations:** ^1^ Department of Laboratory, Shandong Daizhuang Hospital, Jining, China; ^2^ Molecular and Experimental Surgery, University Clinic for General-, Visceral-, Vascular- and Trans-Plantation Surgery, Medical Faculty University Hospital Magdeburg, Otto-von Guericke University, Magdeburg, Germany

**Keywords:** machine learning, endocrine disorders, diagnosis, treatment, artificial intelligence

The endocrine system is an important regulatory system in the human body. It regulates physiological processes such as growth, development, metabolism, and reproduction through the secretion, transfer, and feedback of hormones. Various factors, including environmental, genetic, and lifestyle factors, can lead to the development of endocrine diseases associated with dysfunction of the pituitary, adrenal, and thyroid glands, as well as diabetes. Changes in living environments and lifestyles have led to steady increases in the incidence of endocrine diseases. If not detected and treated in time, these can lead to complications and cause irreversible damage to health.

The application of machine learning in medical fields has great prospects (1). This Research Topic is the use of machine learning to establish models for the prediction, diagnosis, and management of endocrine-related diseases for the benefit of both patients and clinical practice. The following is a brief overview of some of the included articles:

The model built by Wang et al. using the Gaussian Naive Bayes (GNB) algorithm allows improved prediction of prolonged hospitalization of patients following acute ischemic stroke (AIS). This will assist in policy adjustments for improved resource utilization, thereby alleviating the increasingly heavy economic burden caused by AIS.

Chen et al. used a two-sample Mendelian randomization (MR) study to explore the relationship between thyroid function and cholelithiasis. Their findings showed that low-density lipoprotein cholesterol (LDL-C) and apolipoprotein B mediated the effects of FT4 on cholelithiasis risk, with patients with high FT4 levels showing delayed or reduced risk of the long-term effects of cholelithiasis.

Zeng et al. analyzed data from the Genomics of Drug Sensitivity in Cancer (GDSC) and The Cancer Genome Atlas (TCGA) databases, using Spearman correlation analysis to identify the key genes that influence Gemcitabine (GEM) efficacy. It was found that *CALB2* and *GPX3* could be used as biomarkers for prognosis prediction in some forms of colorectal cancer (CRC) as well as potential target genes of GEM, providing new ideas for the development of new combined targeted drugs for colorectal cancer.

Liu et al. established a machine learning-based choice for some type 2 diabetic kidney disease(T2DKD) risk prediction model based on clinical data from a multi-center retrospective database and verified its effectiveness. While the model was found to be helpful for the diagnosis of T2DKD, further investigation using additional data is required.

Huang et al. used the ESTIMATE algorithm to conduct a series of bioinformatics analyses on breast cancer (BC) samples from the TCGA database to identify genes associated with the tumor microenvironment (TME). The authors found a significant correlation between KLRB1 and the BC TME, suggesting its use as a prognostic marker and therapeutic target, providing a new direction for the treatment of BC.

Li et al. identified stratified prognostic biomarkers for serous ovarian cancer (SOC) after investigation of immune infiltration, drug sensitivity, and genes associated with the epithelial-mesenchymal transition (EMT). This lays a foundation for in-depth investigation of the role of the EMT in SOC immune regulation and changes in related pathways. It also suggests effective solutions for the early diagnosis and clinical treatment of ovarian cancer.

Xu et al. identified the C11 cluster that specifically expresses HSPB6 in fibroblasts as key to the development of pancreatic adenocarcinoma (PAAD). The authors used comprehensive bioinformatics analyses and constructed a nine-gene prognostic model using tumor-related PAAD prognostic genes in the C11 subgroup. The RiskScore may have reliable clinical potential for the prognostic prediction of PAAD.

With the increased informatization of society and further accumulation of data, the use of machine learning will become more common in medical applications requiring the processing of large amounts of data. Machine learning can process large amounts of data that humans cannot handle and is a powerful and versatile tool for future medicine (2). The application of machine learning in the diagnosis and treatment of endocrine diseases will become increasingly widespread and mature, as is currently seen in the prediction, diagnosis, and management of diseases such as diabetes (3), thyroid disease (4), and neuroendocrine tumors (5).

## Author contributions

HZ: Formal analysis, Investigation, Methodology, Resources, Writing – original draft. UK: Conceptualization, Data curation, Project administration, Resources, Writing – review & editing. WS: Conceptualization, Investigation, Methodology, Project administration, Resources, Supervision, Writing – review & editing.
